# Magnetic resonance imaging follow-up can screen for soft tissue changes and evaluate the short-term prognosis of patients with developmental dysplasia of the hip after closed reduction

**DOI:** 10.1186/s12887-021-02587-2

**Published:** 2021-03-08

**Authors:** Xianghong Meng, Jianping Yang, Zhi Wang

**Affiliations:** 1 Department of Radiology, Tianjin Hospital, Jiefangnan Road, Hexi District, Tianjin, 300211 TJ China; 2 Department of Orthopedic Pediatrics, Tianjin Hospital, Jiefangnan Road, Hexi District, Tianjin, 300211 TJ China

**Keywords:** Developmental dysplasia of the hip, Closed reduction, Magnetic, Resonance imaging, Residual acetabular dysplasia, Femoral head necrosis

## Abstract

**Background:**

Magnetic resonance imaging (MRI) can show the architecture of the hip joint clearly and has been increasingly used in developmental dysplasia of the hip (DDH) confirmation and follow-up. In this study, MRI was used to observe changes in the hip joints before and after closed reduction (CR) and to explore risk factors of residual acetabular dysplasia (RAD).

**Methods:**

This is a prospective analysis of unilateral DDH patients with CR and spica cast in our hospital from October 2012 to July 2018. MRI and pelvic plain radiography were performed before and after CR. The labro-chondral complex (LCC) of the hip was divided into four types on MRI images. The variation in the thickening rate of the ligamentum teres, transverse ligaments, and pulvinar during MRI follow-up was analyzed, and the difference in cartilaginous acetabular head index was evaluated. The “complete relocation” rate of the femoral head was analyzed when the cast was changed for the last time, and the necrotic rate of the femoral head was evaluated after 18 months or more after CR. Lastly, the risk factors of RAD were analyzed.

**Results:**

A total of 63 patients with DDH and CR were included. The LCC was everted before CR and inverted after CR, and the ligamentum teres, transverse ligaments, and pulvinar were hypertrophic before and after CR, and then gradually returned to normal shape. The cartilaginous acetabular head index gradually increased to normal values. Complete relocation was observed in 58.7% of femoral heads, while 8.6% had necrosis. The abnormalities in LCC was related to RAD (OR: 4.35, *P* = 0.03), and the rate of type 3 LCC in the RAD group was higher. However, the IHDI classification (*P* = 0.09); the “complete relocation” of femoral heads (*P* = 0.61); and hypertrophy of the ligamentum teres (*P* = 1.00), transverse ligaments (P = 1.00), and pulvinar (P = 1.00) were not related to RAD.

**Conclusions:**

In this study, MRI can observe the variations of the abnormal soft tissue structures of the diseased hips after CR and spica casting, and can evaluate which hips will have RAD after CR. Therefore, we can utilize MRI in DDH patients appropriately.

## Background

Developmental dysplasia of the hip (DDH) is one of the most common musculoskeletal disorders in children, and some patients may develop hip osteoarthritis in early adulthood and already require joint replacement [1]. The treatment of DDH is determined by the age at initial diagnosis, degree of hip dislocation, and initial therapeutic effects. Patients with DDH between 6 and 24 months or with failed Pavlik harness treatment within 6 months are treated with either conservative or surgical treatment modalities, such as closed reduction (CR) with spica casting and femoral osteotomies. CR and spica casting are the most utilized conservative treatment [2, 3].

The patient needs an intraoperative X-ray arthrography to evaluate whether CR is successful and to detect abnormal soft tissue structures in the hip, such as hypertrophied acetabular cartilage, narrowed capsule, and labral inversion [4, 5]. A plain pelvic film was typically used for follow-up after CR. However, X-ray films cannot evaluate the alignment of the acetabulum and the femoral head accurately nor observe the changes in soft tissue structures of the hip directly [6]. Magnetic resonance imaging (MRI) has no radiation and can clearly show the soft tissue and osseous structures of the hip joint, and its use in DDH detection and follow-up has been increasing [7–9].

Currently, it is debated whether the abnormal soft tissue structures in the hip of DDH patients can hinder CR and affect the outcome. Renshaw et al. [10] found that “false reduction”, where reduction was achieved immediately after CR but eventually the hip joint became unstable due to obstruction of soft tissue structures, can occur in some patients. However, Druschel et al. [11] believed that abnormalities of soft tissue structures did not affect the success of CR.

Initially, it is often impossible to obtain a concentric reduction in the affected hip after CR. Complete relocation is achieved when the femoral head reaches the bottom of the acetabulum [12]. Some authors believe that complete relocation may increase the risk of femoral head necrosis, which may be caused by increased pressure in the hip joint after reduction or due to damage to the blood supply of the femoral head when performing CR [13]. In addition, risk factors for residual acetabular dysplasia (RAD) after CR are also being studied extensively. Some authors believe that the cartilaginous/osseous acetabular index, cartilaginous/osseous central-edge angle, the shape of the acetabular load area, and the abnormal high signal intensity on T2WI of acetabular cartilage are risk factors for RAD [14, 15].

In this study, MRI was used to observe changes in the soft tissue structure of the hip joints of DDH patients undergoing CR. This study also aimed to explore whether complete relocation is a risk for femoral head necrosis, and to identify the risk factors of RAD after CR.

## Methods

Tianjin Hospital Ethics Committee approved the study (Approval number: 2017医伦审018). Informed consent was obtained from all the parents of individual participants included in the study. The datasets used and/or analysed during the current study are available from the corresponding author on reasonable request.

### Inclusion and exclusion criteria

In this prospective study, DDH is diagnosed if the osseous acetabular index (OAI) > 30° [1]. The study enrolled patients aged 6–24 months with unilateral developmental hip dislocation from October 2012 to July 2018. The exclusion criteria are: 1) CR failure when performing initial MRI, and re-dislocation at the time of replacing spica casts; 2) poor image resolution or poor body position leading to difficulties in diagnosis; 3) hip dysplasia caused by neuromuscular abnormalities, such as Ehlers-Danlos disease, congenital torticollis, equinovarus, arthrogryposis, and achondroplasia. A total of 78 patients met the inclusion criteria; six patients were excluded due to CR failure, four patients due to re-dislocation, three patients due to poor image resolution, and two patients due to other neuromuscular abnormalities. Therefore, 63 patients (63 hips) with hip dislocation were included in this study.

### Imaging examinations

Patients were examined by a plain pelvic film before and after CR, and the degree of DDH was classified according to the International Hip Dysplasia Institute (IHDI) classification [16]. All patients were examined by arthrography before CR under general anesthesia. Using an 18^#^ needle, 0.5–1 mL of iohexol was injected into the hip joint space under the long adductor tendon to observe soft tissues in the hip. Reduction was performed using gentle Ortolani manipulation. The hips were fixed at flexion (90–110°) and abduction (55°) by spica casts; if the maximal abduction angle was < 60°, adductor tenotomy was performed. Some patients underwent pelvic MRI examination 1 month before CR, and all patients underwent pelvic MRI examinations within 24 h after CR and for follow-up before changing spica casts. Each patient underwent MRI three to four times. All MRI examinations were performed on a 3.0 T MR scanner (MR750, GE Healthcare, Milwaukee, WI, USA) with an eight-channel cardiac coil. Because the patients were too young to cooperate, diluted chloral hydrate, prepared by dissolving 1 g of chloral hydrate in 10 mL of normal saline, was injected into the anus 30 min before the MRI examination. The injection volume of the diluted chloral hydrate was 0.5 mL/kg. The patient laid in a supine position, with the lower extremities naturally straightened and the patella facing forward; the scan range was from the iliac crest to the femoral lesser trochanter. Routine MRI protocols and parameters of the imaging sequences are described in Table 1. After CR and spica casting, the patients wore a temporary night abduction brace for 3–12 months based on the development of the diseased hips. They also underwent pelvic X-ray examination every half year after CR to observe the development of the diseased hips. If a patient > 5 years old developed RAD, we performed surgical treatment at a proper time to resolve the RAD.

**Table 1:** Routine sequences and parameters of MRI examinations in the study

Sequence	TR (ms)	TE (ms)	Bandwidth (kHz)	FOV (cm)	Slice thickness (mm)	Slice gap (mm)	Matrix	NEX
Coronal FS PDWI	2500	40	85	22 × 22	3.5	0.5	320 × 224	3
Transverse FS PDWI	2500	40	63	22 × 22	3.5	0.5	320 × 256	3
Coronal T2WI	3000	85	85	24 × 16	3.5	0.5	320 × 224	6

*MRI* magnetic resonance imaging, *TR* repetition time, *TE* echo time, *FOV* field of view, *NEX* number of excitations, *TA* acquisition time, *FS* fat suppression, *PDWI* proton density-weighted imaging

### Observation and measurements

The patients changed spica casts once or twice within 4–6 months after CR and were followed up for at least 18 months.

**Fig. 1:**
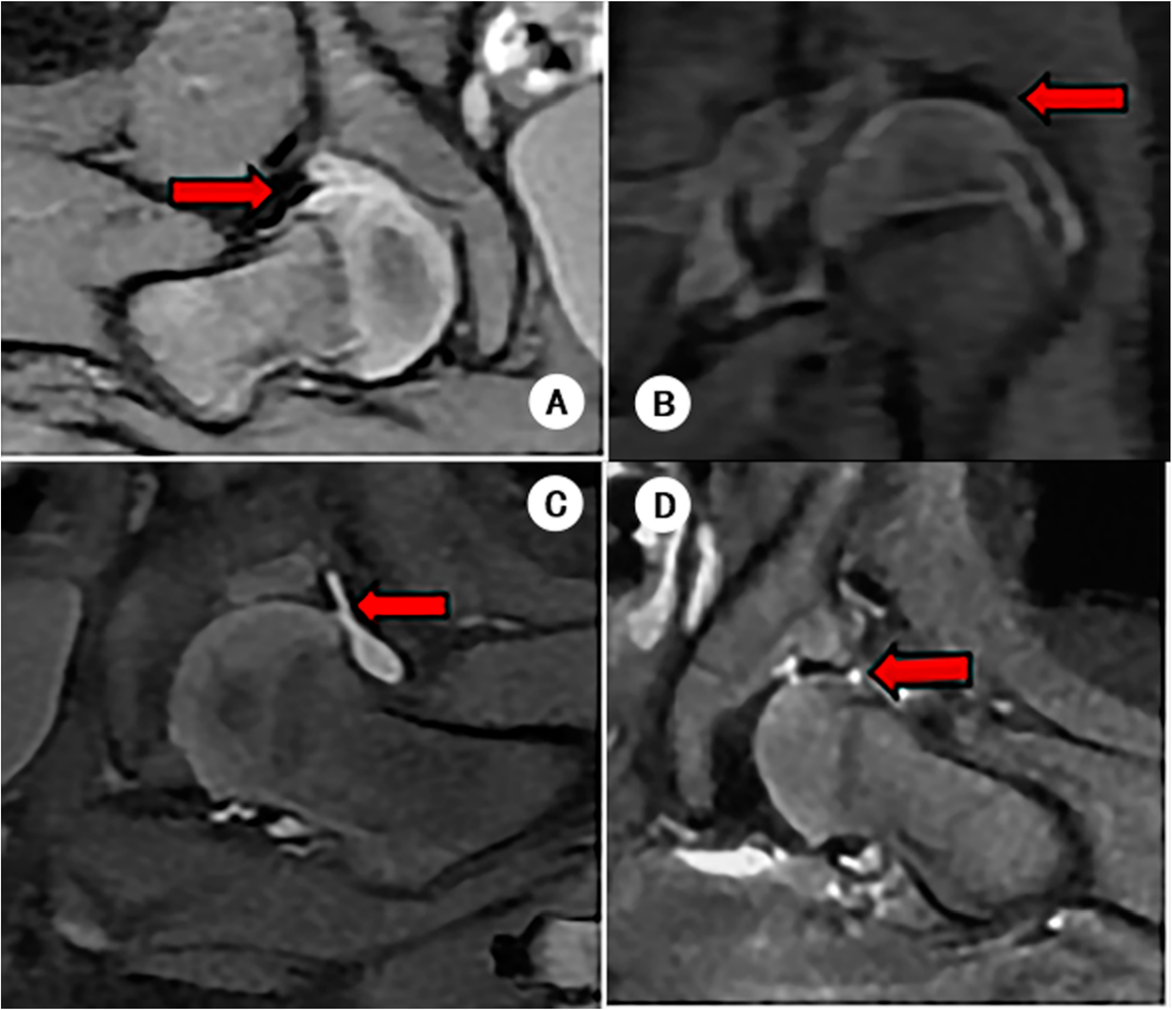
The labro-chondral complex classification. The labro-chondral complex was divided into 4 types (Coronal FS PDWI images), type 1 (**a**): normal (acetabular cartilage and labrum matches, the morphology of labrum is triangular and outward); type 2 (**b**): everted (mild hypertrophy of acetabular cartilage, the labrum is round and outward); type 3 (**c**): partially inverted (mild or moderate hypertrophy of the acetabular cartilage, the labrum inverted partially, which inserts between the femoral head and the acetabulum); type 4 (d): completely inverted (the labrum is inverted entirely and with significant cartilage hyperplasia)

In our study, the labro-chondral complex (LCC) was classified into four types: type 1: normal (acetabular cartilage and labrum matches, the morphology of the labrum is triangular and outward); type 2: everted (mild hypertrophy of acetabular cartilage, the labrum is round and outward); type 3: partially inverted (mild or moderate hypertrophy of the acetabular cartilage, the labrum inverted partially, which inserts between the femoral head and the acetabulum); type 4: completely inverted (the labrum is inverted entirely and there is significant cartilage hyperplasia) (Fig. 1).

**Fig. 2:**
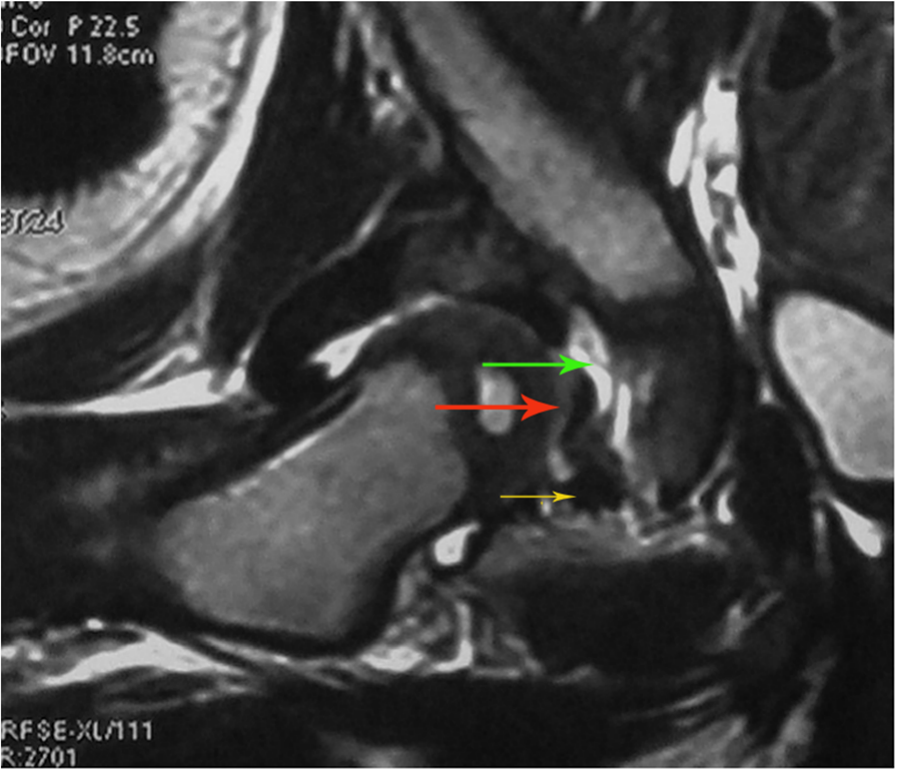
Hypertrophy of the ligamentum teres, the transverse ligament, and the pulvinar. Hypertrophy of the ligamentum teres (red arrowhead) and the transverse ligament (yellow arrowhead) (Coronal T2WI images): the ligament was thicker than that in the normal side, the morphology of the ligament was strip-like, tortuous, prolonged, and manifested mixed signal intensity; pulvinar hypertrophy (green arrowhead): the pulvinar was thick and visible

The study observed whether the ligamentum teres, the transverse ligament, and the pulvinar hypertrophied before and after CR. Hypertrophy of the ligamentum teres and the transverse ligament: the ligament was thicker than that on the normal side, and was strip-like, tortuous, prolonged, and manifested mixed signal intensity; pulvinar hypertrophy: the pulvinar was thick and visible (Fig. 2).

**Fig. 3:**
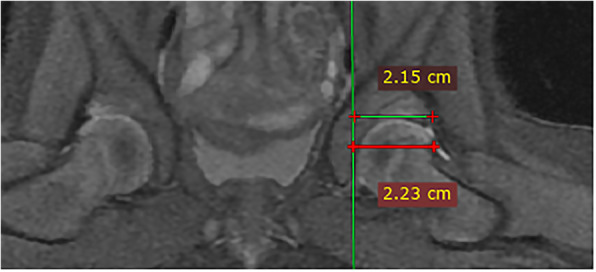
Measurement of cartilaginous acetabular head index. Measurement of cartilaginous acetabular head index (CAHI) of the affected hip in a DDH patient after close reduction and spica casting: on the FS PDWI coronal image showing the maximal diameter of the femoral head, a vertical line was drawn from the innermost edge of the femoral head cartilage, the distance between the line and a line perpendicular to the outermost edge of the acetabular cartilage was measured, and another distance between the line and a line perpendicular to the outermost edge of the femoral head cartilage was also measured. The ratio of the two distances was the CAHI (2.15÷2.23 × 100 = 96.4)

The study also measured the cartilaginous acetabular head index (CAHI) [17] of the affected hip. On the coronal sequence of fat suppressed proton density weighted imaging, the image displaying the maximal diameter of the femoral head was selected, a vertical line was drawn from the innermost side of the femoral head cartilage, and the distance between the line and a line perpendicular to the outermost side of the acetabular cartilage was measured. Another distance between the line and a line perpendicular to the outermost side of the femoral head cartilage was also measured. The ratio between the two distances was the CAHI (Fig. 3).

The complete relocation rate of the femoral head at the affected side during the last replacement of spica casts was calculated on MRI images. On transverse and coronal MRI images, complete relocation was accomplished when the inner edge of the femoral head of the affected side completely contacts the acetabular bottom without soft tissue structures interspersed between them [18]. The presence of femoral head necrosis in the affected hip was determined according to the Salter classification on plain radiography [19].

The OAI of the affected hip was measured on a pelvic plain film during the last follow-up, and patients with OAI > 25° were considered to have RAD [20]. The patients were divided into the normal acetabular group and the RAD group.

All observations and measurements were completed by one radiologist (MXH, with 10 years of musculoskeletal MRI experience). Another radiologist (WZ, with 29 years of musculoskeletal MRI experience) also classified the LCC in the affected hips, and MXH re-classified the complex 2 weeks after her first classification.

### Statistical analysis

The study used the SPSS software (version 25.0; SPSS Inc., Chicago, IL, USA) for statistical analysis. The counting data were expressed as a percentage, while the measurement data were expressed as the mean ± standard deviation. Reliability and repeatability of LCC classification were evaluated by intraclass correlation coefficient (ICC) and 95% confidential interval (CI). The reliability was low when ICC < 0.5, 0.5 ~ 0.75 was medium, 0.76 ~ 0.9 was good, and > 0.9 was excellent [21]. After classifying the LCC, the two radiologists made the final classification by consensus. LCC classifications were analyzed before and after CR. The trend Chi-square test or Fisher exact probability method was used to analyze whether the hypertrophic rates of ligamentum teres, transverse ligament, and pulvinar in affected hips were different immediately after CR and during follow-up. The repeated measurement data analysis of variance or a Mann-Whitney U test was used to evaluate whether the CAHI of affected hips was different immediately after CR and during follow-up. The complete relocation rate of the affected hip at the last MRI follow-up was summarized. The rate of femoral head necrosis in DDH patients who were followed up for 18 months or more after CR was also summarized. The Mann-Whitney U test was used to compare the age of onset and the follow-up time between the normal acetabular group and RAD group. Binary logistic regression was used to analyze whether the IHDI classification; LCC classification at the last MRI examination; hypertrophy of ligamentum teres, transverse ligament, and pulvinar; and complete relocation were risk factors for RAD, using odds ratios (OR) and 95% confidence interval (CI) to indicate the degree of risk. *P* < 0.05 was considered statistically significant.

## Results

### Patient data

A total of 63 patients (63 hips) with CR and spica cast were included in this study, including two boys, 61 girls, 24 right hips, and 39 left hips, with an average age of 15.6 ± 4.4 months (6–23 months). Regarding the IHDI grade, two hips were classified as grade 2, 37 were classified as grade 3, and 24 were classified as grade 4. The average time between CR and the first spica cast change was 70.8 ± 14 (45–100) days, and 55 patients changed spica casts twice. The average time between the first and second spica cast change was 67.8 ± 10.1 (45–101) days. There were 36 patients who underwent MRI before CR, 63 patients underwent MRI immediately after CR and the first spica casts change, and 55 patients underwent MRI at the second time of spica casts change.

### Soft tissue changes and CAHI in the affected hip before and after CR

**Fig. 4:**
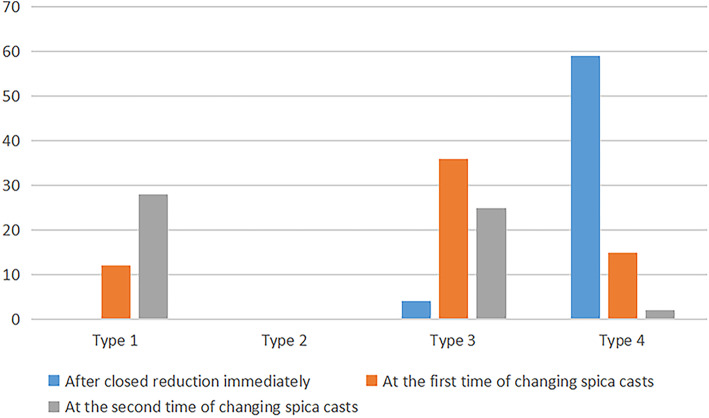
The number and changes in the labro-chondral complex (LCC) type immediately after CR and at the first and the second time of changing spica casts

The inter-observer ICC of the LCC classification between the two radiologists was 0.84 (95% CI: 0.74 ~ 0.91), and the intra-observer ICC was 0.94 (95% CI: 0.90 ~ 0.97). The number and changes of the LCC type after CR and at the first and second spica casts change are listed in Fig. 4. The LCC gradually returned to its normal shape. All patients had hypertrophied ligamentum teres, transverse ligament, and pulvinar in the affected hips immediately before and after CR. About 70% returned to normal for the first time of changing spica casts, and 90% returned to normal at the second time of changing spica casts. The detailed data are presented in Table 2. For the patients who changed spica casts twice (55 patients), CAHI had differences among the time after CR, the first spica cast change, and the second spica cast change (F = 68.0, *P* = 0.000). The CAHI was 68.1 ± 12.1 immediately after CR, increased to 81.2 ± 7.5 when changing casts for the first time, and 84.4 ± 7.0 in the second spica casts change. For the patients who only changed the casts once (eight patients), the CAHI was 60.1 ± 11.1 immediately after CR, and 81.4 ± 6.4 when changing the casts; the CAHI was significantly increased (Z = -3.15, *P* = 0.002).

**Table 2:** Hypertrophic rates of the affected hips in DDH patients

	Hypertrophy rate of ligamentum teres(%)	Hypertrophy rate of transverse ligaments(%)	Hypertrophy rate of pulvinar(%)
**Closed reduction immediately**	96.4%(53/55)	94.5%(52/55)	100%(55/55)
	100%(8/8)	100%(8/8)	100%(8/8)
**First follow up**	27.3%(15/55)	25.5%(14/55)	29.1%(16/55)
	62.5%(5/8)	62.5%(5/8)	62.5%(5/8)
**Second follow up**	12.7%(7/55)	12.7%(7/55)	10.9%(6/55)
**χ**^**2**^ **value (having 3 MRI examinations)**	88.6	86.4	97.9
***P*** **value (having 3 MRI examinations)**	0.000	0.000	0.000
***P*** **value (having 2 MRI examinations)**	0.200	0.200	0.200

### The complete relocation rate and the rate of femoral heads necrosis

MRI images showed that 58.7% (37/63) of the femoral heads have complete relocation at the last time of changing spica casts. For patients who changed spica casts twice, 61.8% (34/55) achieved complete relocation, while for those who changed casts only once, 25% (2/8) achieved complete relocation. A total of 58 patients were followed up after CR for more than 18 months, with an average follow-up time of 39.9 ± 12.8 (18–66) months, and 8.6% (5/58) of the femoral heads had necrosis (Fig. 4). The OAI was 25.0° ± 7.4° (8.2°–40.7°) in the affected hips, of which 48.3% (28/58) of the OAI was no more than 25°, indicating that the osseous acetabulum returned to normal.

### Risk factors of RAD

**Fig. 5:**
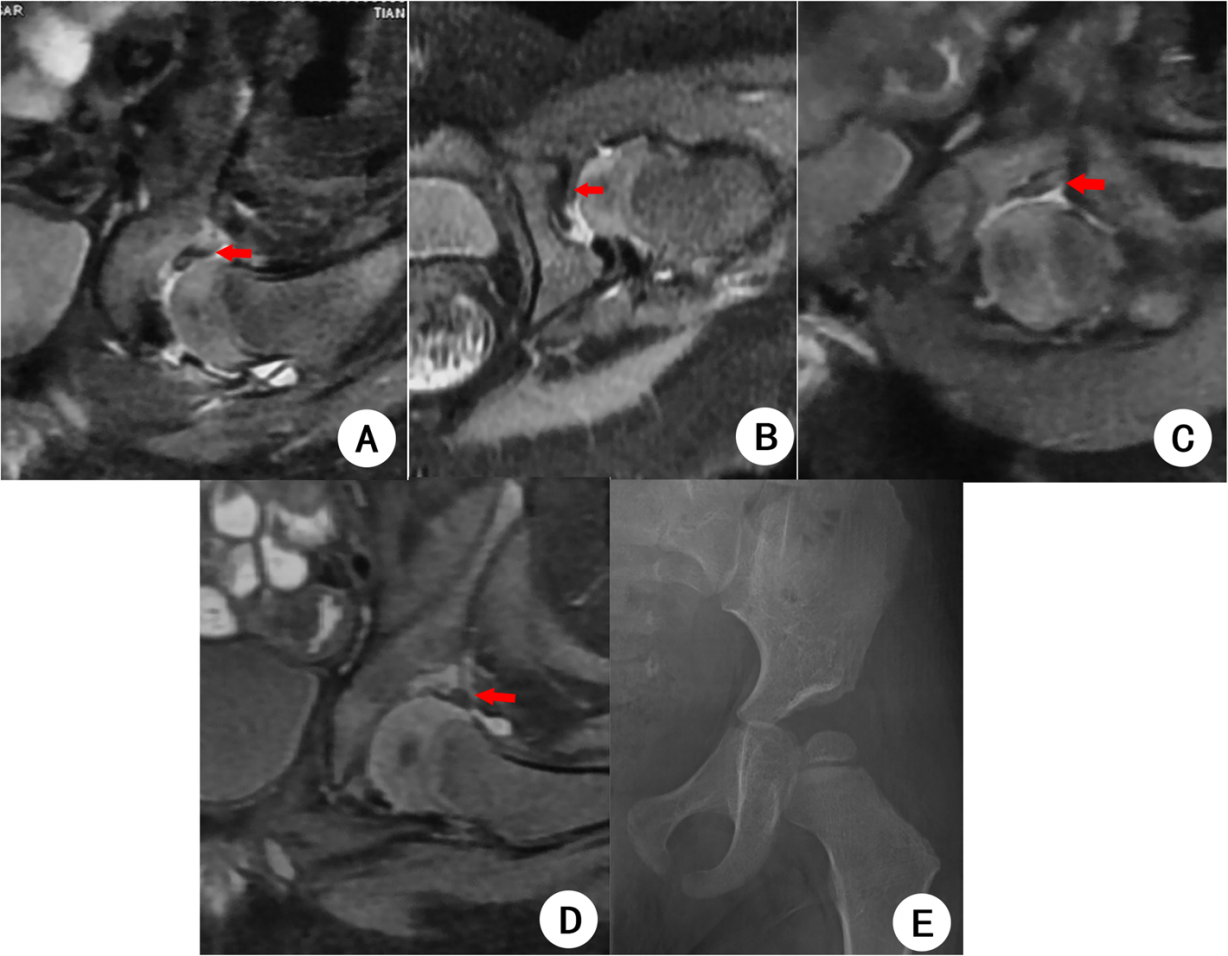
A female patient 10 months after closed reduction and spica casting of the left hip joint. The patient underwent MRI examination of both hips immediately after CR, 3 months after CR, and five and a half months after reduction. **a** and **b** show the coronal and transverse FS PDWI images of MRI immediately after CR. It is suggested that the LCC of the left hip is hypertrophied and completely inverted immediately after reduction, which is considered as type 4; the ligamentum teres, transverse ligament, and pulvinar are hypertrophic. **c** shows that the LCC became flattened 3 months after reduction, and the ligamentum teres, transverse ligament, and pulvinar were less hypertrophic than before. **d** shows that five and a half months after reduction, the LCC still had partial inversion which was considered as a type 3 complex. The ligamentum teres, transverse ligament, and pulvinar were not thickened and returned to normal, and the left hip joint achieved complete relocation. **e** shows the pelvic X-ray film of the patient 22 months after closed reduction, the left osseous acetabular index is 33.3°, indicating that there is still residual acetabular dysplasia; the shape of the left femoral head is intact, and no obvious abnormal density is found

Of the 58 patients who were followed up after CR for more than 18 months, eight patients changed their spica casts only once, and the MRI follow-up time was relatively short, so these eight patients were excluded. Among the remaining 50 patients, 23 patients with OAI > 25° were included in the RAD group (Fig. 5), and 27 patients with OAI ≤ 25° were included in the normal acetabular group. There was no significant difference in the age of onset between the two groups, and the follow-up time in the RAD group was shorter than in the normal acetabular group as shown in Table 3. Binary logistic regression analysis showed that the morphological abnormalities of LCC at the second time of changing spica casts were related to RAD (OR: 4.35, 95% CI: 1.15 ~ 16.46, *P* = 0.03), and the percentage of type 3 LCC was higher in the RAD group. However, there was no significant difference in the IHDI grade before CR (*P* = 0.09); complete relocation at the second MRI examination after CR (*P* = 0.61); and the hypertrophic rates of ligamentum teres (*P* = 1.00), transverse ligament (P = 1.00), and pulvinar (P = 1.00) between the two groups.

**Table 3:** Parameters between the residual acetabular dysplasia group and normal acetabular group

	Age of onset (month)	Follow-up time (month)
**Residual acetabular dysplasia group**	16.1 ± 4.2	36.1 ± 12.7
**Normal acetabular group**	14.7 ± 4.9	45.3 ± 11.8
***Z*** **value**	−0.695	−2.426
***P*** **value**	0.487	0.015

## Discussion

The study found that after 4–6 months of CR and spica casting, the ligamentum teres, the transverse ligament, the pulvinar, and the LCC in the affected hip joint gradually returned to normal shape, and 61.8% of the femoral head had complete relocation. The patients who followed up for more than 18 months revealed that the rate of the femoral head necrosis caused by CR was about 8.6, and 48.3% of the OAI returned to normal. Hypertrophy and partial inversion of the LCC 4–6 months after CR were risk factors for RAD, while hypertrophic ligaments, pulvinar, and femoral head complete relocation had nothing to do with RAD.

In this study, LCC was classified based on MRI images. The results show that this classification has good consistency, high reliability, and repeatability, and can be used in everyday work. Before CR, the labra were everted, and during CR, the labra were caught up in the hip joint with the reduction of the femoral head, resulting in labral hypertrophy and inversion. Therefore, most of the LCC cases were type 4 and a few were type 3 immediately after CR. With the gradual inward displacement of the femoral head after reduction and complete relocation, the LCC also gradually changes shape, the labra gradually grow outward with acetabular cartilage thinning, and it returned to the normal shape. For type 2 LCC, the everted labrum is more common in patients with subluxation of the hip joint before CR. The labrum is everted because of the outward and upward displacement of the femoral head, which is rarely seen after CR.

There are many studies on soft tissue structures in and around the hip joint, which hinder CR in DDH patients [22–24]. Studer et al. [22] observed that hypertrophy of the ligamentum teres, transverse ligaments, pulvinar, joint capsule, inverted labrum, and acetabular cartilage hypertrophy were important factors that hindered CR. Rosenbaum et al. [25] concluded that labral hypertrophy and inversion, and hypertrophy of the pulvinar, ligamentum teres, and transverse ligaments were the main reasons hindering CR. By arthrography and MRI, Hattori [24] and Kim [26] found that obvious soft tissue insertion in the hip joint, widening of the medial contrast cistern, and LCC hypertrophy would increase the probability of CR failure, thus increasing the need for open surgery, even if surgical treatment cannot achieve a good prognosis. Yuan et al. [27] found that poor delineation of the labrum and acetabular surface during arthrogram predicted failure of CR in children with DDH, and medial dye pool distance ≥6 mm significantly increased the risk of CR failure. However, the studies by Severin [12] and Aoki [28] have shown that the inverted labrum can be gradually shaped and returned to a normal shape after CR without affecting the final acetabular-head alignment. Walter et al. [29] believed that the hypertrophy of soft tissues in the hip joint does not lead to CR failure. The failure is due to the mismatch between the femoral head and the acetabulum. Lü et al. [30] found that if the LCC was thin, most hips could be successfully reduced and achieve complete relocation, while patients with thick LCC would prevent reduction of the femoral head. In our study, the incidence of hypertrophy of the pulvinar, ligamentum teres, and transverse ligaments was high before and at the time of CR, but these structures gradually returned to normal. Therefore, the authors believe that the abnormal soft tissue structures of the affected hip joints at reduction have no significant effect on the ultimate outcome, and it seems that it is not necessary to deal with these structures at the time of reduction. The CAHI of the affected hips increased gradually after CR, suggesting that CR increases the stress between the femoral head and the acetabulum and the cartilaginous acetabulum develops.

This study found that at the second time of replacing spica casts (4–6 months after CR), most femoral heads can achieve complete relocation, meaning that the hip joint achieved concentric reduction. One of the most serious complications of CR is secondary femoral head necrosis. The cause of necrosis is unknown, but may be related to the interruption of blood supply to the femoral head, excessive abduction of the hip joint, and increased stress on the femoral head. It is suggested that the rate of femoral head necrosis after CR in patients with DDH varies between 0 and 67% [13, 31]. This study found that the rate of femoral head necrosis with 18 months or more of follow-up after CR was 8.6%, which was acceptable and were lower than the multicenter study of Li et al. [3]. It was shown that with the improvement of orthopedic surgeons’ understanding of the “safe zone” when performing hip abduction, CR and spica casting proved to be a safe and effective treatment for 6–24-month-old DDH patients.

In this study, it was found that partial inversion of the labrum at 4–6 months after CR was a risk factor for RAD, while complete relocation of the femoral head does not influence the development of osseous acetabulum. Some risk factors of RAD have been found [32]; however, these factors are still controversial, and there is a lack of in-depth research on the causes of abnormal signals and parameters of acetabular cartilage. We have conducted a study on the application of T2 mapping combined with CUBE [33], which found that the T2 values of acetabular and femoral head cartilage in patients with inverted labra were higher than those in patients without labra inversion, and the more serious the labral inversion, the higher the T2 value, suggesting that the acetabular cartilage in patients with labral inversion was mostly made of hyaline cartilage with increased free water content, and it could not be mineralized in time. Combined with this study, we believe that labral inversion can hinder the normal ossification of the acetabular cartilage, resulting in RAD after reduction. The complete relocation of the femoral head suggests that CR can achieve a concentric reduction of the hip in patients with DDH, but if the labral inversion persists, it will hinder the normal development of acetabular cartilage.

This study has some limitations. First, the sample size in the RAD group and the normal acetabular group included in this study was small, and the diagnostic efficiency was insufficient. It is necessary to further increase the number of patients in each group in the future. Second, the follow-up time of the patients after CR was uneven and relatively short, without follow-up until the patients grew up, and the outcome of the condition of the patients was unknown. The follow-up time of the normal acetabular group is longer than that of the RAD group, so there may be some patients in the RAD whose OAI returned to normal with time. Therefore, it is necessary to continue long-term follow-up in these patients to observe the outcome. Lastly, the use of MRI repeatedly in young children is impractical in the clinical setting because of compliance, need for anesthesia, and expenses; therefore, it is very difficult to perform MRI examinations universally.

## Conclusion

In this study, MRI can observe the variations of the abnormal soft tissue structures of the diseased hips after CR and spica casting, and can evaluate which hips will have RAD after CR. Therefore, we can utilize MRI in DDH patients appropriately.

## Data Availability

Most of the data supporting our findings are contained within the manuscript, and all others will be shared upon request.

## Abbreviations

DDH: Developmental dysplasia of the hip
CR: Closed reduction
MRI: Magnetic resonance imaging
RAD: Residual acetabular dysplasia
OAI: Osseous acetabular index
IHDI: International Hip Dysplasia Institute
LCC: Labro-chondral complex
CAHI: Cartilaginous acetabular head index
ICC: Intraclass correlation coefficient
CI: Confidential interval
